# An Optimal Multi-Channel Trilateration Localization Algorithm by Radio-Multipath Multi-Objective Evolution in RSS-Ranging-Based Wireless Sensor Networks

**DOI:** 10.3390/s20061798

**Published:** 2020-03-24

**Authors:** Xuming Fang, Lijun Chen

**Affiliations:** 1 School of Software Engineering, Jinling Institute of Technology, Nanjing 210000, China; 2 Department of Computer Science and Technology, Nanjing University, Nanjing 210000, China; chenlj@nju.edu.cn

**Keywords:** multi-channel ranging, indoor positioning, multi-objective optimization, wireless sensor networks

## Abstract

The Global Positioning System (GPS) is unable to provide precise localization services indoors, which has led to wireless sensor network (WSN) localization technology becoming a hot research issue in the field of indoor location. At present, the ranging technology of wireless sensor networks based on received signal strength has been extensively used in indoor positioning. However, wireless signals have serious multipath effects in indoor environments. In order to reduce the adverse influence of multipath effects on distance estimation between nodes, a multi-channel ranging localization algorithm based on signal diversity is herein proposed. In real indoor environments, the parameters used for multi-channel localization algorithms are generally unknown or time-varying. In order to increase the positioning accuracy of the multi-channel location algorithm in a multipath environment, we propose an optimal multi-channel trilateration positioning algorithm (OMCT) by establishing a novel multi-objective evolutionary model. The presented algorithm utilizes a three-edge constraint to prevent the traditional multi-channel localization algorithm falling into local optima. The results of a large number of practical experiments and numerical simulations show that no matter how the channel number and multipath number change, the positioning error of our presented algorithm is always smaller compared with that of the state-of-the-art algorithm.

## 1. Introduction

Because the Global Positioning System (GPS) is unable to accurately determine locations indoors, obtaining high-precision localization services in indoor environments has become urgent. To meet this requirement, indoor localization systems based on wireless sensor networks (WSNs) have been developed [1,2,3]. The WSNs are a kind of self-organizing network composed of many nodes with wireless transceivers, microprocessors, and sensors [4,5,6]. The received signal strength indication (RSSI) is the easiest to measure by a wireless transceiver, so it is often used to estimate distances and locations between nodes [7,8,9].

RSSI-based positioning technologies include fingerprint positioning [10], Radar [11], Doppler Frequency Shift (DFS) [12], ranging positioning [13], among others. Fingerprint location first measures the RSSIs of many places to construct a fingerprint map, then uses a fingerprint matching algorithm to determine the location of the target node. In a relatively stable RSSI environment, the positioning accuracy of this method is high. However, in dynamic RSSI environments, the positioning accuracy of this method is not high. Similar to fingerprint location, Radar also determines the location of a target node through a fingerprint map. The difference between the two is that Radar uses a simple linear-time search algorithm to replace the complex fingerprint matching algorithm in a fingerprint location. Like fingerprint location, the positioning accuracy of Radar depends heavily on the stability of RSSI. DFS uses the Doppler effect to determine the location of the moving target. Although DFS is no longer affected by the stability of RSSI, it will be affected by indoor multipath effects. RSSI-based range positioning uses a multi-channel signal propagation model to determine the location of a target, and can adapt to changes in RSSI and suppress multipath effects by fitting model parameters.

At present, in addition to the RSSI-based ranging technique, some ranging technologies without RSSI can also be used for indoor location, such as angle of arrival (AOA), time difference of arrival (TDOA), and time of arrival (TOA) [14,15,16]. TOA calculates the distance between nodes by measuring the time of wireless signal propagation. Similarly, TDOA estimates the distance difference by measuring the time difference of wireless signal transmission to different nodes. Unlike TDOA, AOA estimates the angle by measuring the time difference between wireless signals transmitted to different antennas of the node. Due to the high speed of wireless signal propagation, high-precision time synchronization is required between nodes when using TOA, TDOA, and AOA. For WSNs with limited cost and energy, this is difficult to achieve. Since the RSSI-based positioning algorithm does not require high-precision time information, it is very suitable for resource-constrained WSNs.

Due to the multipath effect of wireless signals, the accuracy of positioning based on RSSI is usually not high in indoor environments. In order to overcome the influence of the multipath effect on the accuracy of RSSI-based localization, the diversity of different channel signals has been employed to improve this accuracy in multipath environments [13,17,18]. The multi-channel localization algorithm utilizes the difference of different channel signals to fit the parameters in the signal propagation model. Because the number of parameters to be fitted is usually larger than the number of equations, and there is the problem of ill-conditioned matrices in the process of fitting, this makes the existing multi-channel localization algorithm fall easily into local optima.

In order to resolve this issue, we present an optimal multi-channel trilateration positioning algorithm (OMCT) applied to WSNs employing a newly built multi-objective evolutionary model. By adding a trilateral constraint objective function, our proposed algorithm can find the global optimal parameter values and prevent the fitting parameter values falling into local optima. The execution of the OMCT algorithm is divided into three steps. First, the RSSI measurement noise is filtered by an adaptive Kalman filter (AKF). Next, the values of two objective functions are calculated using the filtered RSSIs of multiple channels. Lastly, the optimal node position estimate is found by the multi-objective evolutionary algorithm (MOEA).

The following are our main contributions in this study.

(1) On the basis of the three-edge constraint, we create a new multi-objective evolution model. As far as we know, this model is the only one that can prevent the evolved node distance falling into local optima at present.

(2) According to the newly established multi-objective evolutionary model, we present an optimal multi-channel trilateration location algorithm for wireless sensor networks. Unlike the existing multi-channel positioning algorithm, the proposed algorithm solves the problem of the location result depending heavily on the initial value of the parameter.

(3) Through a large number of real experiments and numerical simulations, we verify the efficiency and effectiveness of the presented algorithm. The results of extensive experiments and simulations show that the presented algorithm greatly improves the accuracy of the most advanced multi-channel location algorithm, regardless of whether the initial values of the parameters are accurate or not.

This paper consists of six sections, and its organizational structure is as follows. Section 2 shows the work related to the RSSI-based multi-channel trilateration localization algorithm. Section 3 describes the formalization of the multi-channel positioning optimization problem. Section 4 gives a description of the execution steps of the presented algorithm. Section 5 presents the analysis and results based on experiments and simulations. Finally, Section 6 summarizes this paper.

## 2. Related Work

At present, based on whether the distance between nodes needs to be obtained during positioning, location algorithms in WSNs are classified into two types: algorithms with ranging [19,20] and those without ranging [21,22]. In the location algorithms using ranging, the node’s distance or angle is utilized to estimate the position of the target node. The WSN localization algorithms based on ranging mainly include trilateration [23], triangulation [24], and maximum likelihood estimation [25]. Trilateration uses the distances between one target node and three anchor nodes to locate the target node, while triangulation first converts the angles between three anchor nodes and one target node into the corresponding distances, then estimates the target node’s location through trilateration. Maximum likelihood estimation is also called multilateration and is an extension of trilateration. When the distances between more than three anchor nodes and one target node are obtained, maximum likelihood estimation employs the least square method to calculate the position of the target node.

The typical range-free location algorithms are multidimensional scaling-map (MDS-MAP), distance vector-hop (DV-Hop), centroid, and convex position estimation (CPE) [26,27,28,29]. The CPE algorithm transforms the WSN positioning problem into a convex optimization planning problem. In the process of planning, the rectangular area in which the target node is located is obtained. The centroid algorithm determines whether the target node can be covered by a triangle composed of three anchor nodes, then selects the next triangle to make the same judgment. Finally, the target node’s coordinates are represented by the geometric centroid of the overlap region. The DV-Hop algorithm first calculates the average distance per hop between nodes. Then, the target node’s location is estimated according to the indirect distance by trilateration or maximum likelihood estimation. The MDS-MAP algorithm uses the relative distance and link information between nodes to determine the relative positions in the network based on multidimensional scaling technology.

Although location algorithms without ranging do not require knowing the distance to the node, their accuracy is generally not as high as that of the ranging localization algorithms. At present, the ranging techniques for WSN localization algorithms are mainly TOA, TDOA, AOA, and RSSI. Since RSSI-based location has no requirement for high-precision time synchronization, it is more suitable than TOA, TDOA, and AOA for resource-constrained WSNs. According to the number of channels used in the positioning process, the RSSI-based localization algorithms are divided into two categories: single-channel and multi-channel. The classic RSSI-based single-channel positioning algorithms mainly include Radar, Landmarc, Rips, and ranging location based on the log-normal shadowing model (LNSM) [30,31,32,33]. Radar first constructs an RSSI map of the environment, then looks for the location of the target node by referring to this map. Similarly, Landmarc determines the position of the target node by finding some reference nodes with RSSIs similar to the RSSI of the target node. The fingerprint location method based on radio mapping needs to spend much time measuring the RSSIs in advance of different positions, which is not suitable for an RSSI-changeable environment. Rips uses the exact distance between the nodes obtained by radio interferometry to locate the target node. However, this localization algorithm suffers severe multipath effects, especially in indoor environments. LNSM-based ranging positioning first establishes the model of signal propagation by measuring the RSSIs at different distances, then estimates the location of the target node through the established model. Although this method saves the time and labor of fingerprint location in measuring RSSIs in many locations, as well as facilitating deployment in WSNs, there is a big deviation between the model of RSSI and distance established by it and the relationship between RSSI and distance in the actual environment, especially in a multipath environment. Aside from LNSM, other models have been used to describe the relationship between RSSI and distance, such as the free space propagation model (FSPM) [34] and the two-ray ground reflection model (TGRM) [8]. FSPM establishes the relationship between RSSI and distance when a wireless signal propagates in open space, so it does not consider the reflection effect of the ground and obstacles on the signal. Although TGRM considers the reflection effect of a ground surface on the signal, it does not consider the reflection effect of other obstacles on the signal. Therefore, there are still large errors in the ranging positioning methods based on these two signal propagation models in a multipath environment.

To reduce the influence of multipath effects on the accuracy of single-channel positioning algorithms, multi-channel localization algorithms based on multipath cancellation technology have been proposed. The algorithm proposed by Llizaro et al. uses the frequency diversity of signals of multiple channels to suppress the negative influence of multipath effects on the location results of RSSI-based positioning algorithms [17]. However, this algorithm simply averages the RSSI values of different frequencies without modelling the signal relationship between different paths. Zhang D. et al. proposed an RSSI-based ranging localization algorithm distinguishing the multiple radio paths, namely, a multipath-distinguishing-based trilateration positioning algorithm (MUDT) [13]. This algorithm estimates the distance between nodes through a newly established multipath signal propagation model for different channels, the positioning accuracy of which depend heavily on the initial values of the model parameters. The main reason for this is that this algorithm fits the model parameters based on the least square method, and the parameter number is greater than the equation number, so the fitted parameters easily fall into a local optimum when the initial values of the parameters are closer to a local minimum rather than the global minimum. Zhang C. et al. proposed a training-free RSSI ranging localization algorithm for WSNs [18]. This algorithm eliminates the multipath effect by weighting the RSSIs measured in different channels, but it needs to know the distance between a certain reference node and the target node in advance.

## 3. Problem Formalization

In this section the system model is introduced and the formalization of the problem is described. The basic idea of a multi-channel localization algorithm is to acquire an estimation of the target node’s location by collecting the measured values of different RSSI channels.

In the system model, it is assumed that the transmitter and receiver can dynamically adjust the transmission frequency between them, and that each receiver/transmitter pair can cooperate and synchronize well. At present, this function can be supported by most off-the-shelf WSN products.

Suppose there are *n* signal transmission paths between a given receiver/transmitter pair. Without loss of generality, take the first path as the Line-Of-Sight (LOS) path and the rest as Non-Line-Of-Sight (NLOS) paths. The lengths of these paths are represented by *d_i_*, i=1,…,n. $\rho_{i}$ is the reflection coefficient of path *i*, where $\rho_{1}$ of the LOS path is equal to 1, and those of the NLOS paths are less than 1. Assume that the RSSI values on *m* different channels can be measured from the target node and the anchor one; $\lambda_{j},j=1,\ldots ,m$ are the signal wavelengths corresponding to the *m* channels.

For a synthetic signal with different paths, the RSSI measured at the receiver is a vector sum [13]. On the *j*-th channel, the synthetic signal strength formed by *n* different paths is expressed as

(1) $$P\left(x,\lambda_{j}\right)=\left({\left(\sum_{i=1}^{n}\rho_{i}c\lambda_{j}^{2}d_{i}^{-2}\sin\left(d_{i}\lambda_{j}^{-1}\right)\right)}^{2}\right.{\left.+{\left(\sum_{i=1}^{n}\rho_{i}c\lambda_{j}^{2}d_{i}^{-2}\cos\left(d_{i}\lambda_{j}^{-1}\right)\right)}^{2}\right)}^{1/2},$$

where $x=\left(c,\rho_{2},\ldots ,\rho_{n},d_{1},\ldots ,d_{n}\right)$, $c=\frac{P_{t}G_{t}G_{r}}{{\left(4\pi \right)}^{2}}$, *P_t_* is the transmission power between nodes, *G_t_* is the transmitter’s antenna gain, and *G_r_* is the receiver’s antenna gain.

Suppose the actual RSSI measured on channel *j* is expressed as *P_j_*, j=1,…,m. The optimization goal of multi-channel trilateration localization based on the RSSI is to find an optimal position of the target node and make the fitted synthetic signal strength $P\left(x,\lambda_{j}\right)$ and real measurement *P_j_* as close as possible.

## 4. The Proposed Algorithm

### 4.1. The Existing AKF

In an actual environment, the measurement noise of the RSSI is often unknown and time-varying. An inaccurate RSSI noise parameter will seriously reduce the positioning accuracy of the proposed algorithm. In this subsection, an adaptive Kalman filter (AKF) that can sense the noise parameter is described [35]. Through adding a noise estimator to the Kalman filter, an AKF is created. The AKF algorithm is completed based on the following three steps.

**Step 1.** Prediction:

(2) $${\hat{x}}_{k\left|k-1\right.}=f\left({\hat{x}}_{k-1}\right),$$

(3) $$F_{k}=\left.\frac{\partial f}{\partial x}\right|\begin{array}{c} {\hat{x}}_{k-1} \end{array},$$

(4) $$P_{k\left|k-1\right.}=F_{k}P_{k-1}F_{k}^{T}+Q_{k},$$

where ${\hat{x}}_{k-1}$ is the observed system’s state estimation for time step *k*−1, ${\hat{x}}_{k\left|k-1\right.}$ is the system state prediction for time step *k*−1, $F_{k}$ is a Jacobian of $f\left(•\right)$ calculated based on ${\hat{x}}_{k-1},$ $P_{k-1}$ is the state estimation’s expected covariance, $P_{k\left|k-1\right.}$ is the covariance of state estimation calculated based on $P_{k-1}$, and **Q***_k_* is the covariance matrix of process noise.

**Step 2.** Correction:

(5) $${\hat{z}}_{k}=h\left({\hat{x}}_{k\left|k-1\right.}\right),$$

(6) $$S_{k}=H_{k}P_{k\left|k-1\right.}H_{k}^{T}+R_{k},$$

(7) $$H_{k}=\left.\frac{\partial h}{\partial x}\right|\begin{array}{c} {\hat{x}}_{k\left|k-1\right.} \end{array},$$

(8) $${\hat{x}}_{k}={\hat{x}}_{k\left|k-1\right.}+K_{k}\left(z_{k}-{\hat{z}}_{k}\right),$$

(9) $$K_{k}=P_{k\left|k-1\right.}H_{k}^{T}S_{k}^{-1},$$

(10) $$P_{k}=P_{k\left|k-1\right.}-K_{k}H_{k}P_{k\left|k-1\right.},$$

where ${\hat{z}}_{k}$ is the estimation of the measurement from the observation system at time step *k*, $S_{k}$ is the covariance expectation of measurement estimation, $H_{k}$ is a Jacobian of $h\left(•\right)$ calculated according to ${\hat{x}}_{k\left|k-1\right.},$ ${\hat{x}}_{k}$ is the calibration of state estimation at time step *k*, $K_{k}$ is the matrix of the Kalman gain, $P_{k}$ is the state estimation’s covariance calibration, and **R***_k_* is the covariance matrix of the measurement noise.

**Step 3.** Noise covariance estimation:

(11) $$R_{k+1}=\left(1-h_{k}\right)R_{k}+h_{k}\left[\varepsilon_{k}\varepsilon_{k}^{T}-H_{k}P_{k\left|k-1\right.}H_{k}^{T}\right],$$

(12) $$\varepsilon_{k}=z_{k}-{\hat{z}}_{k},$$

(13) $$h_{k}=\left(1-b\right)/\left(1-b^{k+1}\right),$$

where $\varepsilon_{k}$ is the difference between the actual measurement and the measurement estimation at time step *k*, b is a forgetting factor that ranges from 0.95 to 0.995 and is often assigned a value of 0.96, and $h_{k}$ is also a forgetting factor.

### 4.2. The Novel Multi-Objective Evolutionary Model

In this subsection we introduce the multi-objective evolutionary algorithm (MOEA) for the OMCT. Then, a multi-objective evolutionary model is presented that employs objective functions 1 and 2 to evolve the optimal position estimation of the target node. 

An outstanding MOEA for problem optimization is applied to the discovery of the node positions with the best-matched RSSIs, because it does not need to set accurate initial values of node positions in advance [36,37]. Unlike other problem optimization algorithms, such as the least square method, this algorithm can obtain the global optimal solution, and does not easily fall into local optimal solutions. The MOEA algorithm is subdivided into the following four steps.

**Step 1**. Initialize one population ***P****_t_* with *N* individuals randomly, then use genetic operators (mutation, crossover, and tournament selection) to generate one offspring population ***O****_t_*.

**Step 2**. Merge ***P****_t_* with ***O****_t_*, and create one compound population ***I****_t_* with 2*N* individuals. Next, acquire discrepant non-dominated fronts through sorting ***I****_t_* based on non-domination.

**Step 3**. Fill the non-dominated fronts into the next-round population ***P****_t_*_+1_ with *N* individuals.

**Step 4**. If the number of evolution generations does not exceed the maximum value set in advance, the algorithm continues.

Objective functions, variable constraints, and decision variables are used in the MOEA’s multi-objective evolutionary model. Without loss of generality, its form is as follows:

$$minimize\;:\;y=g\left(x\right)=\left(g_{1}\left(x\right),\ldots ,g_{v}\left(x\right)\right),\;subject\;to\;:e\left(x\right)=\left(e_{1}\left(x\right),\ldots ,e_{w}\left(x\right)\right)\leq 0,$$

where *y* is an objective vector containing the values of *v* objective functions, x is a decision vector consisting of decision variables, and *e*(***x***) is a set of *w* constrained conditions of the decision vector.

Objective function 1 in the multi-objective evolutionary model is defined to evolve the multipath RSSI estimate matched with the multi-channel RSSI measurement:

(14) $$g_{1}\left(x\right)=\sum_{l=1}^{3}\sum_{j=1}^{m}{\left(P\left(x_{l},\lambda_{j}\right)-P_{l,j}\right)}^{2},l=1,\ldots ,3,j=1,\ldots ,m,$$

where $P\left(x_{l},\lambda_{j}\right)$ represents the multipath RSSI estimation on the *j*-th channel between the target node and the *l*-th anchor node:

(15) $$\begin{array}{l} P\left(x_{l},\lambda_{j}\right)=\left({\left(c_{l}\lambda_{j}^{2}d_{l,1}^{-2}\sin\left(d_{l,1}\lambda_{j}^{-1}\right)+\sum_{i=2}^{n}\rho_{i}c_{l}\lambda_{j}^{2}d_{l,i}^{-2}\sin\left(d_{l,i}\lambda_{j}^{-1}\right)\right)}^{2}\right.\\ {\left.+{\left(c_{l}\lambda_{j}^{2}d_{l,1}^{-2}\cos\left(d_{l,1}\lambda_{j}^{-1}\right)+\sum_{i=2}^{n}\rho_{i}c_{l}\lambda_{j}^{2}d_{l,i}^{-2}\cos\left(d_{l,i}\lambda_{j}^{-1}\right)\right)}^{2}\right)}^{1/2} \end{array}$$

The result of objective function 1 is the sum of the squares of differences between RSSI measurements and RSSI estimates. The smaller the result of the function, the more accurate the parameter of the evolution.

In order to find the node position, estimate with the best-matching RSSI; the second objective function of the multi-objective evolutionary model is defined as follows:

(16) $$g_{2}\left(x\right)=\sum_{l=1}^{3}\left|S\left(r,u_{l}\right)-d_{l,1}\right|.$$

$S\left(r,u_{l}\right)$ represents the distance between the target node and the *l*-th anchor node:

(17) $$S\left(r,u_{l}\right)=\sqrt{{\left(r_{1}-u_{l,1}\right)}^{2}+{\left(r_{2}-u_{l,2}\right)}^{2}},l=1,\ldots ,3,$$

where $r=\left(r_{1},r_{2}\right)$ is the coordinates of the target node, and $u_{l}=\left(u_{l,1},u_{l,2}\right)$ is the coordinates of the *l*-th anchor node.

The multi-objective evolutionary model is created using objective functions 1 and 2, and is used to search for the optimal position estimation with the best-matched RSSI:

$$minimize\;:\;y=g\left(x\right)=\left(g_{1}\left(x\right),g_{2}\left(x\right)\right),\;subject\;to\;:\min x\leq x\leq \max x,\;where\;:\;x=\left(c_{l},\rho_{l,2},\ldots ,\rho_{l,n},d_{l,1},\ldots ,d_{l,n},r_{1},r_{2}\right),l=1,\ldots ,3.$$

### 4.3. The Proposed OMCT

**Figure 1:**
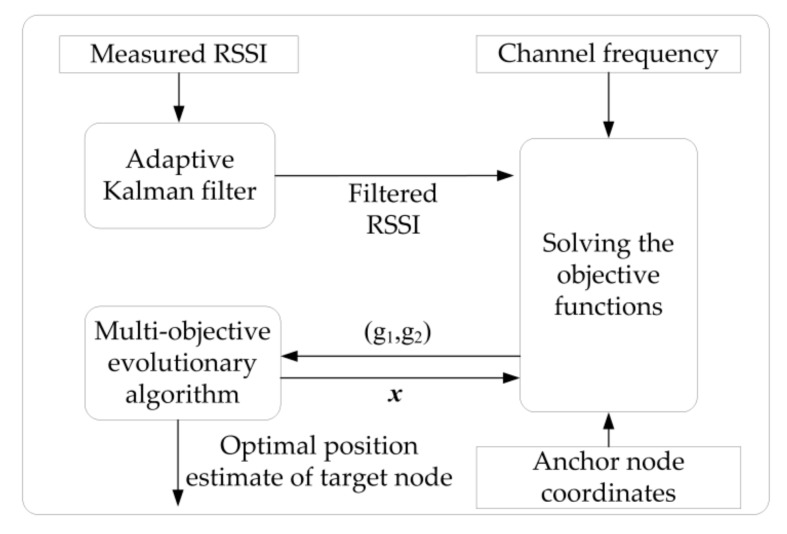
The structure of the optimal multi-channel trilateration positioning algorithm (OMCT) using the received signal strength indication (RSSI).

The multi-objective evolutionary model is employed to obtain the optimal node position estimate by minimizing the calculation result of the objective function. Figure 1 reveals the functional modules of the OMCT algorithm. First, the AKF module is applied for noise reduction of the RSSI measurement. Then, the objective function calculation module obtains the matching degree between RSSI estimations and RSSI measurements based on the filtered RSSI. Lastly, the MOEA module is employed to evolve the node position according to the matching degree. Objective function 1 measures the matching degree between estimations and measurements of multi-channel RSSIs by calculating the square sum of the RSSI differences of different channels. The smaller the calculated value, the closer the evolved multi-channel RSSI is to the actual multi-channel RSSI measurement. Objective function 2 calculates the difference between the distance from the evolved target node to the anchor node and the distance estimated by objective function 1, then measures their matching degree. The smaller the calculated value, the better the evolved distances will match.

There is no conflict between the two objective functions. When they reach the minimum value at the same time, the global optimal parameter value is obtained. However, there are too few constraints among the parameters in the multi-channel signal propagation model, and it is difficult for a single-objective evolutionary algorithm to obtain accurate parameter values. We add a trilateral constraint as the second objective function to exclude inaccurate parameter values. Therefore, the multi-objective evolutionary algorithm with two objective functions is used.

Algorithm 1 gives the implementation steps of the OMCT algorithm.
**Algorithm 1: OMCT****Input:** measured RSSI z, channel frequency *f*, anchor node coordinates ***u*****Output:** optimal target node coordinates $\hat{r}$1. Set the parameters of MOEA2. Initialize AKF3. Utilize AKF to filter z and acquire filtered RSSI $\tilde{z}$
4. Compute signal wavelength λ based on *f*5. Generate *N* decision vectors x by MOEA6. t←1
7. $t_{\max}\leftarrow \mathrm{total}\;\mathrm{number}\;\mathrm{of}\;\mathrm{generations}$
8. **while**
$t\leq t_{\max}$
**do**9.      Calculate *N* g_1_ according to x, $\tilde{z}$ and λ
10.      Compute *N* corresponding g_2_ via x, u
11.      Produce new *N*
x by MOEA based on g_1_ and g_2_12.      t←t+1
13. **end while**14. Select *r* of first x in Pareto front with ascending g_1_ as $\hat{r}$
15. **return**
$\hat{r}$


## 5. Performance Evaluation

In this section, we report the results of a large number of experiments and simulations conducted to evaluate the performance of the proposed algorithm. The results of experiments and simulations are shown under mismatched multipath numbers, actual channel diversity, matched multipath numbers, and simulated channel diversity.

### 5.1. Experimental Setup

In each experiment, eight target nodes and three anchor nodes were stochastically deployed in a 10 × 10 m area; all the nodes were based on the TelosB platform working in the 2.4G Industrial Scientific Medical (ISM) band with 16 channels (i.e., channel 11 to 26), the frequencies of which are shown below:

(18) $$f_{i}=f_{0}+\left(i-11\right)\Delta f,\;i=11,\ldots ,26,$$

where *f_i_* is the frequency of the *i*-th channel in MHz, Δf is the channel interval of 5 MHz, and *f*_0_ is the channel frequency constant, which in this case was 2405 MHz.

The target node switched channels once every 0.1 s to send a message to its neighboring anchor nodes. The three nearest-anchor nodes that received the message on channels 11 to 16 were employed to acquire an estimate of the target node’s location. 

Table 1 provides the parameters and their corresponding values for the proposed algorithm in the experiments. According to the experimental results, it takes about 1000 generations to evolve the minimum value of the objective function. Consequently, the number of evolution generations was assigned as 1000. The values of the parameters min***x*** and max***x*** were chosen according to those used in the actual experiment [13]. The parameters not given and their values were consistent with the common settings of the MOEA algorithm; changes in these had little effect on the location results of the proposed algorithm.

The state transfer function and measurement function for the AKF are composed of unit matrices. The dimension of the matrix is the same as the number of anchor nodes participating in positioning. Since the nodes transmitting and receiving signals are stationary, the process noise covariance of the AKF was set to diag(0,…,0), where the symbol diag represents the diagonal matrix. Since the AKF has the ability to perceive noise, in the case of unknown RSSI measurement noise, the initial measurement noise covariance was set to diag(3,…,3), according to [38]. Because the state estimation error covariance of the AKF has a self-correcting ability during state iteration, its initial value is usually set to diag(1,…,1). In each experiment, the AKF ran the same number of time steps, (i.e., 10).

**Figure 2:**
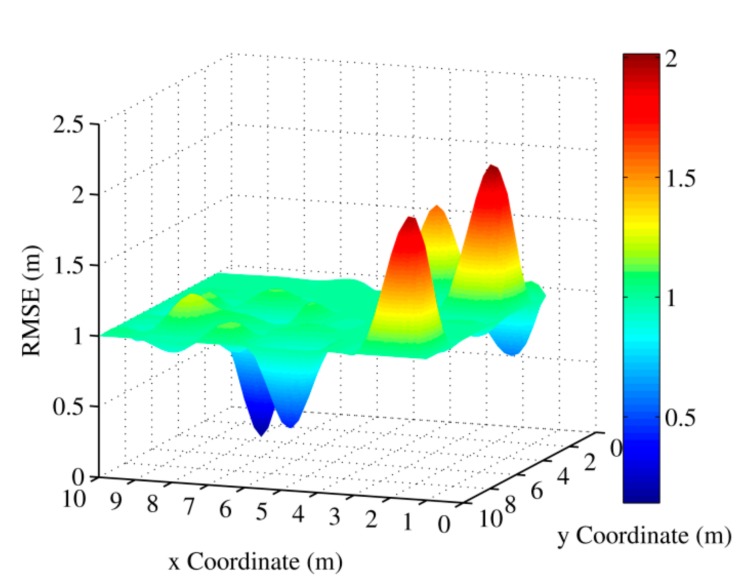
The root-mean-square errors (RMSEs) of OMCT in different positions.

The accuracy of all algorithms was evaluated through the root-mean-square error (RMSE) between the estimated location and the actual one in this paper. Figure 2 shows the error distribution of the OMCT algorithm in different positions when the channel number was 16, the multipath number was 2, and the coordinates of the anchor nodes were (2,0), (10,3), and (1,10).

### 5.2. The Impact of Mismatched Multipath Numbers

**Figure 3:**
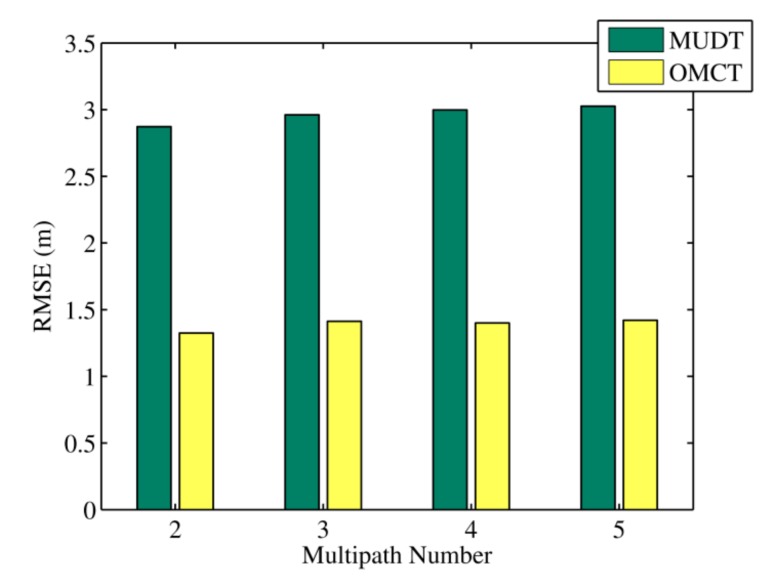
The RMSEs of the multipath-distinguishing-based trilateration positioning algorithm (MUDT) and OMCT under different multipath numbers in the experiment.

The average localization errors of the MUDT and OMCT algorithms after 640 trials under different evolutionary multipath numbers are depicted in Figure 3; these errors were 2.87, 1.32, 2.96, 1.41, 2.99, 1.40, 3.02, and 1.41 m. During the experiments, the number of actual signal propagation paths was unknown. Therefore, the assumed evolutionary multipath number did not in general match the true number of paths. Because the signal strength vector sum of the non-direct paths was equivalent for the multipath signal propagation model, the multi-channel positioning algorithm could also be used to estimate the direct path length between nodes under the condition that the established evolutionary multipath number did not match the actual number of paths. As shown in the figure, the localization errors of the OMCT algorithm were still 53.8%, 52.2%, 53.3%, and 53.1% less than those of the MUDT algorithm, even when the set evolutionary multipath number was inaccurate.

### 5.3. The Impact of Irregular Channel Diversity

**Figure 4:**
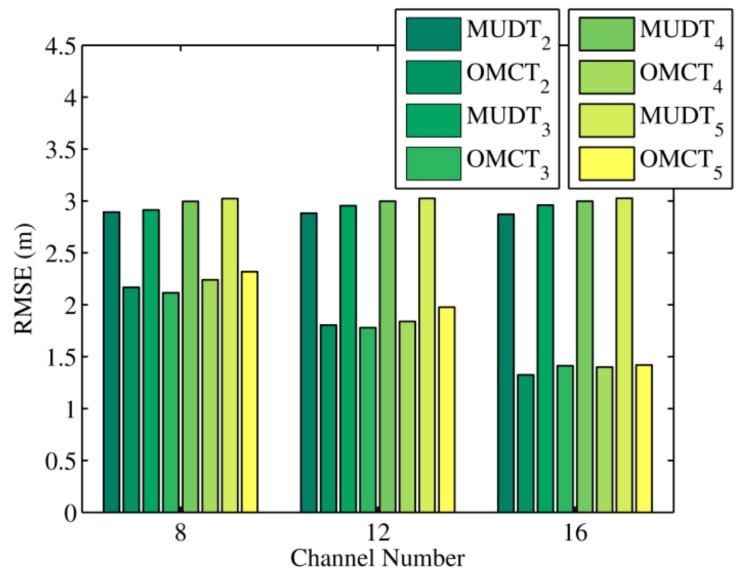
The RMSEs of MUDT and OMCT under different channel numbers in the experiment.

Figure 4 presents the average positioning errors of the MUDT and OMCT algorithms under different channel numbers that were calculated on the basis of the results of 1920 experiments. In the real experiments, the diversity of the multi-channel signal strength was irregular relative to channel strength diversity, which was simulated based on the multipath signal propagation model. In order to observe the effect of different channel diversities on the location result of the evaluated algorithm, we set the numbers of channels to 8, 12, and 16, respectively. As can be seen in the figure, compared with the simulation result, irregular channel diversity increased the deviations in node position estimations by the MUDT and OMCT algorithms. However, since our proposed algorithm increased the constraint relationship between evolutional variables, no matter how the channel number changed its positioning error was always smaller than that of the existing algorithm.

### 5.4. Simulation Setup

In each simulation, three anchor nodes were randomly placed in a 10- x 10-m region, and 10 target nodes were unevenly distributed in this region. The target nodes sent signals to the anchor nodes in 16 channels sequentially. The frequency parameters of the 16 channels were derived from the widely adopted 2.4G ISM band for WSNs.

Because the simulation configuration was the same as that for the experiment, the values of parameters for the MOEA algorithm were consistent with those set in the experiment. The measurement noise covariance of the RSSI was assumed to be 3 according to [38], so the initial measurement noise covariance for the AKF was assigned as diag(3,…,3). The values of other parameters for the AKF were also the same as those used in the experiment.

### 5.5. The Impact of Matched Multipath Numbers

**Figure 5:**
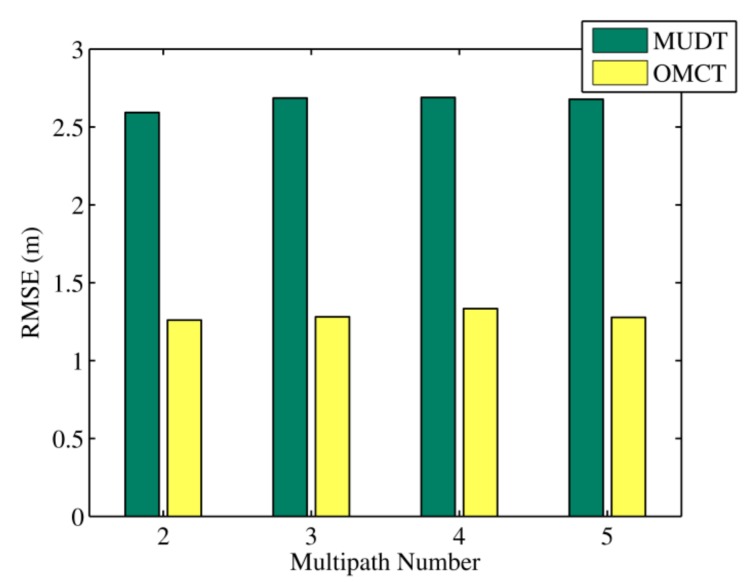
The RMSEs of MUDT and OMCT under different multipath numbers in the simulation.

In Figure 5, the mean location errors of the MUDT and OMCT algorithms under different multipath numbers from 800 simulations are exhibited; these were 2.59, 1.25, 2.68, 1.28, 2.69, 1.33, 2.67, and 1.27 m. Because the number of propagation paths of the simulated multipath signal was known, the multipath number for the multi-objective evolution model could be accurately set; that is, the actual number of signal propagation paths was matched with the evolved multipath number. From the figure, it can be seen that the localization errors of the OMCT algorithm were 51.3%, 52.2%, 50.4%, and 52.3% less than those of the MUDT algorithm when the multipath number equaled 2, 3, 4, and 5, respectively. It follows that no matter how we change the number of evolutionary paths, the presented algorithm always has a higher accuracy than the existing algorithm.

### 5.6. The Impact of Regular Channel Diversity

**Figure 6:**
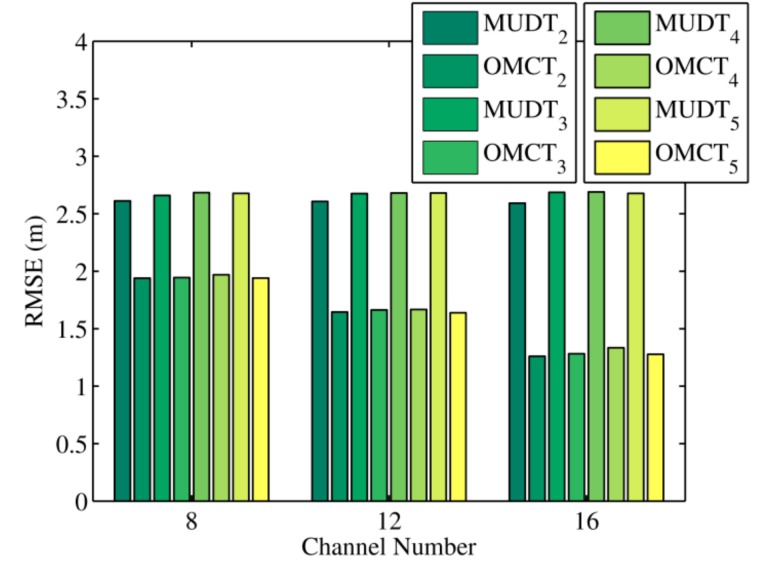
The RMSEs of MUDT and OMCT under different channel numbers in the simulation.

Figure 6 reveals the average localization errors of the MUDT and OMCT algorithms under different channel numbers, which were obtained by executing 2400 simulations. The multi-channel positioning algorithm uses the diversity of signals to estimate the distance and location between nodes, so we decreased the number of channels from 16 to 8 to observe the effect of channel number on the location result of the evaluated algorithm. Because the assigned values were known for all parameters for the multipath signal in the simulation, the diversity of simulated signals was relatively regular. From the figure we can see that when the number of channels decreased, the positioning error of the OMCT algorithm increased. However, even under such unfavorable conditions, the localization error of the OMCT algorithm was still smaller than that of the MUDT algorithm. The main reason for this is that our proposed algorithm increased the three-edge constraint, so the evolved distance estimation between nodes was less trapped in local optima.

### 5.7. Time Complexity Analysis

In this paper, all algorithms were evaluated using MATLAB for the experiments and simulations, and run on a computer with a 2.2 GHz processor; the average execution time was calculated based on the total running time in the experiments and simulations. Since the MUDT algorithm did not need to evolve the position estimate of the target node, its average computing time was about 15 ms. The OMCT algorithm needed to acquire the target node’s optimal location through an evolution of 1000 generations, so its average execution time was approximately 490 ms. Although the time complexity of the OMCT algorithm was increased, its positioning accuracy was always better than that of the MUDT algorithm. The AKF performed very quickly at each step for the OMCT algorithm, so its execution time per time step was only about 1 ms. The time complexity of OMCT was *O*(*MN*^2^) [36], where *M* is the number of objective functions, and *N* is the population size, so we reduced the calculation time of OMCT by selecting a small population size [37]. To sum up, the presented algorithm can meet the real-time positioning requirement of WSNs.

## 6. Conclusions

In this paper, an optimal multi-channel localization algorithm based on trilateration was proposed and employed to improve target node location estimation accuracy under multipath environments. The precision of the existing multi-channel positioning algorithm is not high. The main reason for this is that the distance estimation between nodes easily falls into local optima. To solve this problem, we built a novel multi-objective evolutionary model. In this model, a three-edge constraint was utilized to avoid the distance estimation falling into local optima. In order to test the influence of different multipath numbers and different channel numbers on the accuracy of the multi-channel localization algorithm, we performed numerous experiments and simulations on the proposed and existing algorithms. The experimental and simulation results show that when compared to the existing algorithm, the positioning error of our proposed algorithm is always smaller, regardless of whether the number of multipaths is matched or whether the channel diversity is regular.

## Figures and Tables

**Table 1:** The parameters and values in the multi-objective evolutionary algorithm (MOEA).

Parameters	Values
Population strength	10
Maximum generation	1000
min*c*	0
max*c*	0.00001
min*ρ*	0
max*ρ*	0.3
min*d*	0 m
max*d*	10 m
min*r*_1_	0 m
max*r*_1_	10 m
min*r*_2_	0 m
max*r*_2_	10 m
